# hnRNPA2B1 recognizes RNA virus SFTSV infection through mitochondrial DNA

**DOI:** 10.1128/mbio.01668-25

**Published:** 2025-07-21

**Authors:** Xin-bo Huang, Yue Zhang, Jing-wen Fan, Xin-yu Yu, Yun-lan Fang, Jin-xin Ren, Bin-yan Liu

**Affiliations:** 1 Center for Environment and Health in Water Source Area of South-to-North Water Diversion, School of Public Health, Hubei University of Medicine 74765, Shiyan, Hubei Province, People's Republic of China; 2 Shiyan Key Laboratory of Virology, Hubei University of Medicine 74765, Shiyan, Hubei Province, People's Republic of China; 3 Clinical Skills Teaching and Training Center of Hubei University of Medcine, Shiyan, Hubei Province, People's Republic of China; 4 The Second Clinical School, Hubei University of Medicine 74765, Shiyan, Hubei Province, People's Republic of China; 5 The First Clinical School, Institute of Medicine and Nursing, Hubei University of Medicine 74765, Shiyan, Hubei Province, People's Republic of China; Boston University Chobanian & Avedisian School of Medicine, Boston, Massachusetts, USA

**Keywords:** SFTSV, hnRNPA2B1, mtDNA, STING, IFNβ

## Abstract

The heterogeneous nuclear ribonucleoprotein (hnRNPA2B1, hereafter A2B1) was identified as a novel nuclear DNA sensor that mediates antiviral immunity by recognizing viral DNA in the nucleus. However, it remains largely unclear whether A2B1 could function as a nucleic acid pattern-recognition receptor during cytoplasmic RNA virus infection. Severe fever with thrombocytopenia syndrome virus (SFTSV), which causes severe hemorrhagic fever, is a tick-borne RNA virus that poses a serious threat to public health. In this study, we observed that during SFTSV infection, the interaction between A2B1 and SFTSV nucleoprotein (NP) promoted the retention of A2B1 in the cytoplasm. Importantly, the transcription levels of *IFN*β and inflammatory cytokines were decreased with *A2B1* silenced, similar to the expression levels of p-TBK1 and p-IRF3. We observed that cytoplasmic A2B1 was involved in recognizing mislocated mitochondrial DNA (mtDNA) and triggered the STING-TBK1 axis to potentiate antiviral type I interferon responses. Thus, A2B1 could be identified as a novel cytoplasmic DNA sensor and sense RNA virus SFTSV infection by monitoring mislocalized mtDNA, stimulating antiviral immune response. Our study may provide new insights into host defense against RNA virus infections and should be important for clarifying the complex interaction between A2B1 and virus infection.

**IMPORTANCE:**

hnRNPA2B1 (hereafter A2B1) has been identified as a novel DNA sensor for surveillance of infection from DNA viruses. SFTSV is an RNA virus that causes SFTS with a high case-fatality rate of up to 45.7%. Although SFTSV could utilize A2B1 in viral RNA synthesis for proliferation, whether SFTSV can be recognized by DNA sensor A2B1 and initiate innate immune response remains unexplored. Our study illustrates a complex interplay where SFTSV nucleoprotein (NP) seizes the newly synthesized A2B1 in the cytoplasm, which senses leaked mitochondrial DNA, leading to the activation of the STING-TBK1 signaling pathway to promote IFNβ production. These findings reveal the role of nuclear DNA sensor A2B1 in sensing RNA virus SFTSV infection in the cytoplasm and expand the new understanding of A2B1 in innate immunity. By targeting the A2B1-STING axis, we can potentially develop novel antiviral therapies against SFTSV and possibly other RNA viral infections.

## INTRODUCTION

Severe fever with thrombocytopenia syndrome virus (SFTSV), causing severe hemorrhagic fever, is a tick-borne bunyavirus that was first reported in China in 2009 (1), and was subsequently reported in South Korea and Japan (2). Older age is the main target of SFTSV, which exacerbates disease mainly through dysregulation of host immune cells and uncontrolled inflammatory responses, and the mortality rate is as high as 45.7% (2). In the last decade, accumulated studies have highlighted the importance of SFTSV in public health (3–6). Importantly, due to the lack of knowledge on host immunity against SFTSV infection, there are no specific drugs or vaccines available for the clinical therapy of severe fever with thrombocytopenia syndrome (SFTS) (7).

SFTSV is a single-stranded negative-sense RNA virus, and the genome of SFTSV consists of three segments, in which the large (L) segment encodes RNA-dependent RNA polymerase (RdRp), the medium (M) segment encodes glycoprotein, and the small (S) segment encodes nucleocapsid protein (NP) and non-structural protein (NSs) (8). The SFTSV genome is encapsulated by NP and RdRp to form an RNP complex, which is incorporated into the Golgi or ERGIC-originated phagophore by interacting with glycoprotein and then released into the extracellular space via autophagic secretory vesicles (9, 10). The role of NP in the formation of the RNP complex interferes with host interferon production (6, 11). Previous study shows RNA sensor SAFA can be retained by NP in the cytoplasm for mediating antiviral response (12). Meanwhile, NP serves as a novel virulence factor, which assists in escaping from host immunity by inducing TUFM-mediated mitophagy to degrade MAVS (13).

The innate immune response constitutes the first line of host defense against the infection of various viruses. Pattern recognition receptors (PRRs) are of great importance in triggering the type I interferon signal pathway to clear viral infections (14–16), which occurs not only in the cytoplasm and endosome but also in the mitochondria and nucleus. Depending on the cellular localization of different viruses, viruses can therefore be recognized by multicompartmental PRRs (17–19). In 2019, the heterogeneous nuclear ribonucleoprotein A2/B1 (hnRNPA2B1, hereafter A2B1) was identified as a novel nuclear DNA sensor (20). Before that, it was primarily considered an RNA-binding protein that was involved in RNA biology and the transcriptional regulatory network (21–23). Upon infection with the DNA virus, A2B1 recognizes and interacts with viral dsDNA in the nucleus. A2B1 is then homologously dimerized and demethylated at arginine-226 by the arginine demethylase JMJD6, which promotes nucleocytoplasmic translocation of A2B1, to initiate the IFN pathway in a cyclic GMP–AMP synthase (cGAS)-independent but stimulator of interferon genes (STING)-dependent manner. Moreover, A2B1 facilitates m^6^A modification and nucleocytoplasmic trafficking of mRNA to enhance activation of the IFNβ pathway in response to DNA virus infection (20).

As previously reported, it is widely accepted that RNA PRRs retinoic acid-inducible gene I (RIG-I) and melanoma differentiation-associated protein 5 (MDA5) are important for the recognition of SFTSV infection. These cytoplasmic RNA sensors recognize both unique and common RNA virus species (24). Recognition of microbial RNA by RNA sensors in the cytoplasm leads to transcriptional expression of type I interferons and proinflammatory cytokines (24). Furthermore, infection by RNA viruses, such as SFTSV and dengue virus, can also be sensed by the cytoplasmic DNA sensor cGAS, through inducing mitochondrial DNA (mtDNA) leakage into the cytoplasm for cGAS recognition (25, 26). Notably, cGAS-dependent antiviral immunity can be suppressed by SFTSV NP through hijacking cGAS into NP-induced autophagy (25). Besides, other DNA sensors can also interact with RNA viruses and are involved in the induction of the type I interferon-mediated antiviral response, including TLR9 and IFI16 (19, 27–29).

In this study, we explored whether SFTSV can be recognized by DNA sensor A2B1. We discovered that, as unusual as nucleocytoplasmic translocation initiatively, A2B1 can be retained by SFTSV NP in the cytoplasm, recognizes the released mtDNA after mitochondrial dysfunction, and finally activates the STING-TBK1 signaling pathway for antiviral immune response. These findings may reveal potential targets for the development of therapeutic strategies or drugs against SFTSV infection.

## RESULTS

### Knockdown of A2B1 reduces immune responses induced by SFTSV infection

**Fig 1:**
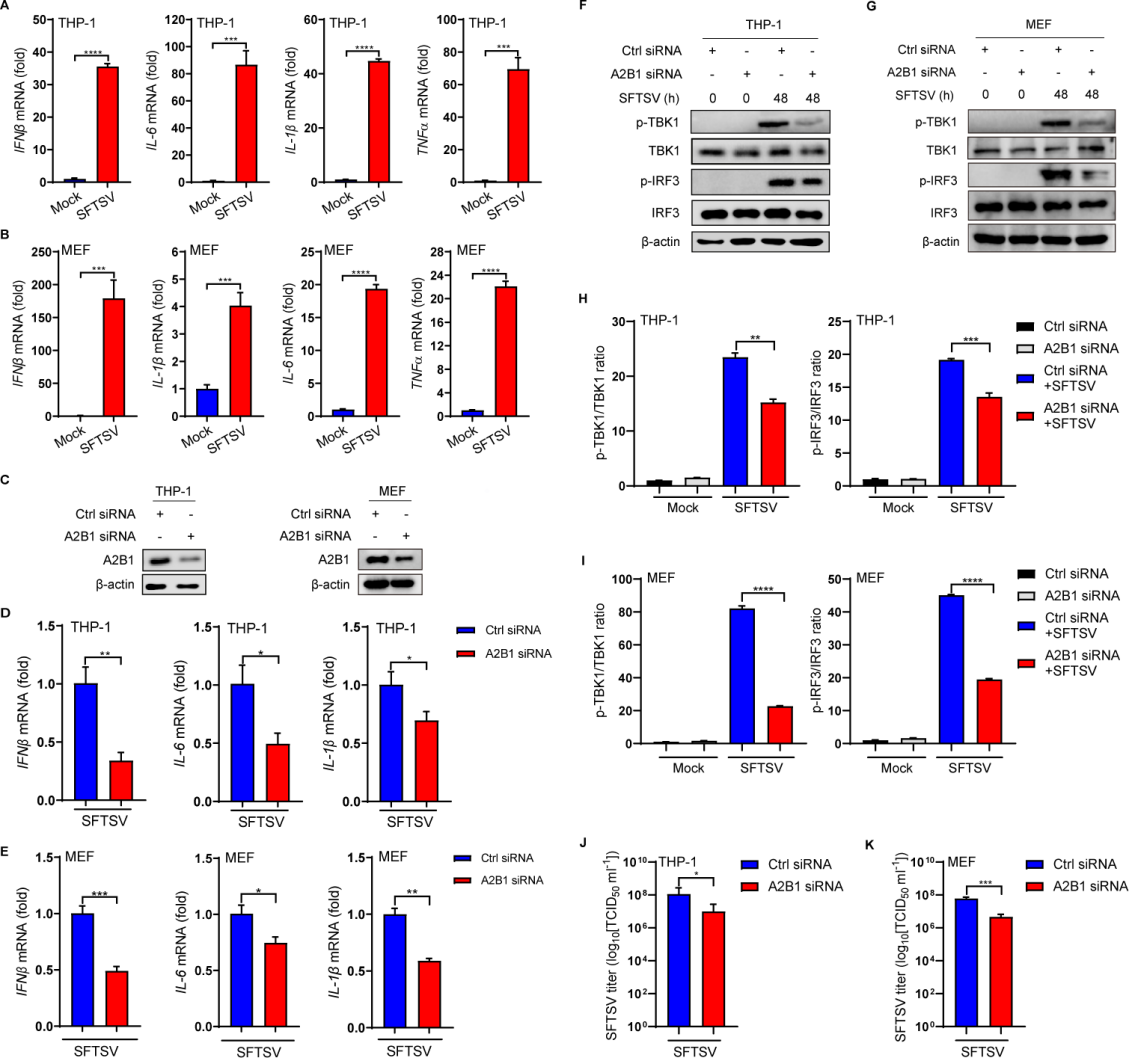
Knockdown of A2B1 reduces IFNβ-dependent STING-TBK1 signaling pathway after SFTSV infection. (**A and B**) THP-1 (**A**) and MEF (**B**) cells were infected with or without SFTSV (MOI = 10) for 48 h. *IFN*β, *IL-6*, *IL-1*β, and *TNF*α mRNA levels were analyzed by qPCR. (**C**) Knockdown of A2B1 in THP-1 and MEF cells, identified by Western blot. (**D and E**) THP-1 (**D**) and MEF (**E**) cells were transfected with A2B1 siRNA (10 µM, 24 h) or Control siRNA (10 µM, 24 h), and then infected with SFTSV (MOI = 10) for 48 h. *IFN*β, *IL-6*, and *IL-1*β mRNA levels were analyzed by qPCR. (**F and G**) THP-1 (**F**) and MEF (**G**) cells were transfected with A2B1 siRNA (10 µM, 24 h) or Control siRNA (10 µM, 24 h), and then infected with SFTSV (MOI = 10) for 48 h. The p-TBK1, TBK1, p-IRF3, and IRF3 protein levels were analyzed by Western blot. (**H and I**) The Western blot data of p-TBK1 and p-IRF3 from panel F (**H**) or G (**I**) were quantified and normalized against TBK1 and IRF3 protein loading control respectively. (**J and K**) THP-1 (**J**) and MEF (**K**) cells were transfected with A2B1 siRNA (10 µM, 24 h) or Control siRNA (10 µM, 24 h), and then infected with SFTSV (MOI = 10) for 48 h. SFTSV titers were measured by TCID_50_ assays. Data were obtained from three independent experiments (*n* = 3). *P < 0.05, **P < 0.01, ***P < 0.001, ****P < 0.0001.

Innate immune cells utilize pattern recognition receptors (PRRs) to detect viruses by recognizing pathogen-associated molecular patterns (PAMPs) and danger-associated molecular patterns (DAMPs) (30). Previous studies have indicated that the protein and mRNA levels of PRRs can serve as indicators for monitoring the activation or suppression of PRRs (30–32). Upon entry into the body, SFTSV dsRNA is recognized by pattern recognition receptors, initiating subsequent signaling cascades. In our study, we used the human macrophage cell line THP-1 and mouse embryonic fibroblast cell line MEF to evaluate the protein and mRNA levels of A2B1 to determine its role in SFTSV infection. We found that the transcription levels of *IFN*β, *IL-6*, *IL-1*β*,* and *TNF*α were significantly increased after SFTSV infection in THP-1 and MEF cells (Fig. 1A and B). To investigate the potential role of A2B1 in antiviral immunity during SFTSV infection, A2B1 siRNAs were utilized in THP-1 and MEF cells to suppress A2B1 expression (Fig. 1C). The mRNA levels of these cytokines were observed to be decreased in THP-1 and MEF cells upon transfection with A2B1 siRNA (Fig. 1D and E), suggesting the potential interaction between A2B1 and antiviral innate immunity during SFTSV infection. The siRNA transfection for 24 h was executed before SFTSV infection for 48 h. The transfection efficiency could be persisted at least for 72 h to ensure the silence of *A2B1* (Fig. S1A and B). And the cell viabilities were not affected during siRNA transfection and SFTSV infection (Fig. S1C through E). In addition, the protein levels of phosphorylated TBK1 (p-TBK1) and phosphorylated IRF3 (p-IRF3) were significantly reduced in A2B1-deficient THP-1 and MEF cells (Fig. 1F and G), and these results were further verified by quantitative analysis (Fig. 1H and I). Furthermore, the median tissue culture infective dose (TCID_50_) was used to detect the exocellular viral titers. We observed that the SFTSV titers were decreased in A2B1 siRNA-transfected THP-1 and MEF cells (Fig. 1J and K). Our data showed that A2B1 deficiency affected the immune function of cells and reduced their ability to respond to SFTSV invasion. These results suggest that A2B1 may play an important role in immunoregulation after SFTSV infection.

### Knockout of A2B1 impairs IFNβ-dependent immune responses against SFTSV infection

**Fig 2:**
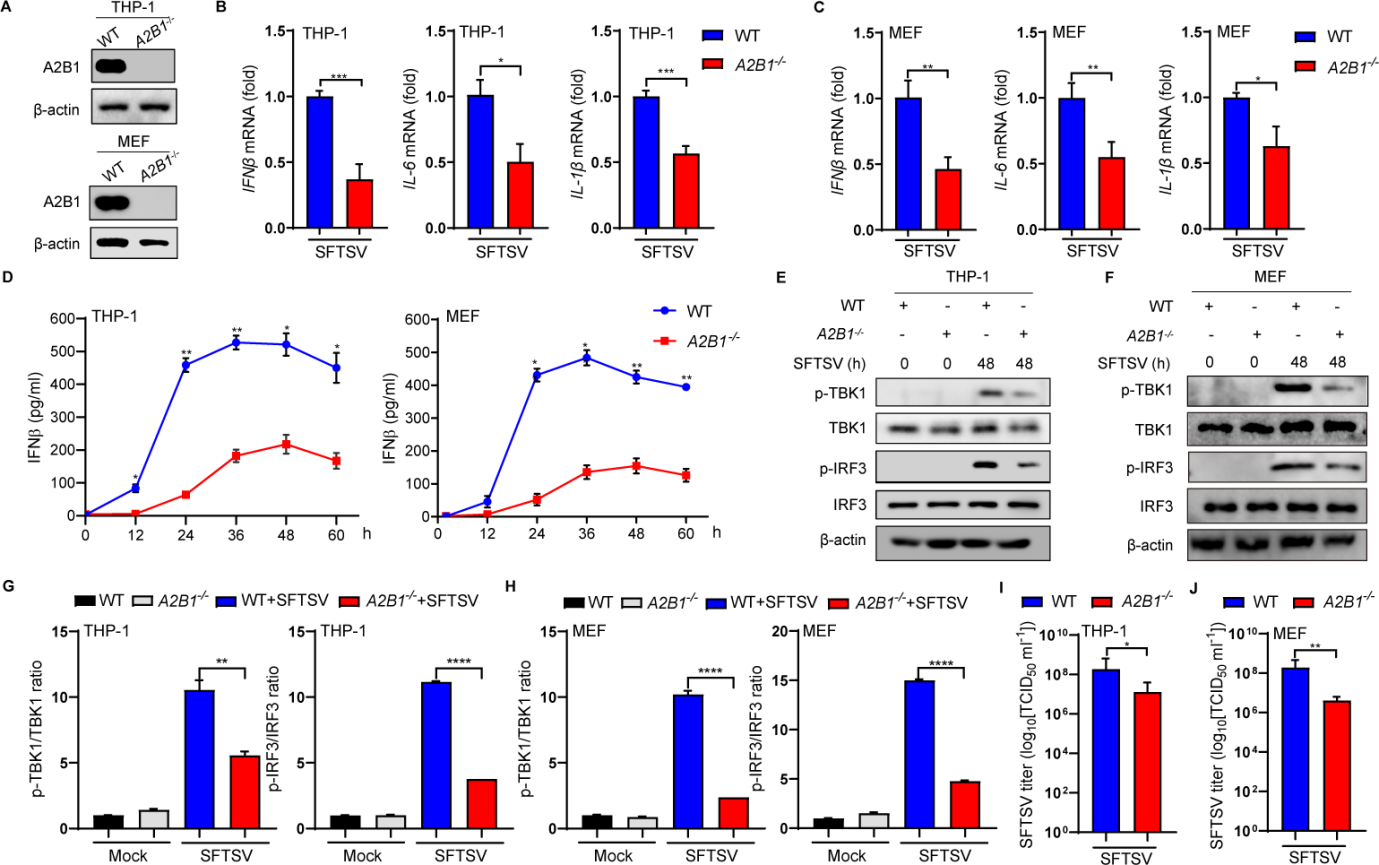
Knockout of A2B1 impairs IFNβ-dependent STING-TBK1 signaling pathway after SFTSV infection. (**A**) Knockout of A2B1 in THP-1 cells and MEF cells, identified by Western blot. (**B and C**) WT/*A2B1*^−/−^ THP-1 (**B**) and MEF (**C**) cells were infected with SFTSV (MOI = 10) for 48 h, *IFN*β, *IL-6*, and *IL-1*β mRNA levels were detected by qPCR. (**D**) WT/*A2B1*^−/−^ THP-1 and MEF cells were infected with SFTSV (MOI = 10) for 48 h, IFNβ cytokine levels were analyzed by ELISA. (**E and F**) WT/*A2B1*^−/−^ THP-1 (**E**) and MEF (**F**) were infected with SFTSV (MOI = 10) for 48 h. p-TBK1, TBK1, p-IRF3, and IRF3 protein levels were analyzed by Western blot. (**G and H**) The Western blot data of p-TBK1 and p-IRF3 from panel E (**G**) or F (**H**) were quantified and normalized against TBK1 and IRF3 protein loading control respectively. (**I and J**) WT/*A2B1*^−/−^ THP-1 (**I**) and MEF (**J**) cells were infected with SFTSV for 48 h. SFTSV titers were measured by TCID_50_ assays. Data were obtained from three independent experiments (*n* = 3). *P < 0.05, **P < 0.01, ***P < 0.001, ****P < 0.0001.

Considering the limitation of the siRNA strategy, A2B1-KO (*A2B1*^−/−^) THP-1 and MEF cells were further constructed by CRISPR/Cas9 system (Fig. 2A). The mRNA levels of *IFN*β, *IL-6*, and *IL-1*β were decreased significantly in *A2B1^−/−^* THP-1 and MEF cells (Fig. 2B and C), suggesting the interaction between A2B1 and antiviral innate immunity during SFTSV infection. Previous studies indicated that the activated STING forms dimers to assemble with TBK1, leading to IRF3 activation and the final induction of type I interferon (33). To further explore the mechanism by which A2B1 functions through the STING-TBK1 pathway, we observed that after SFTSV infection, the transcription of *IFN*β, *IL-6*, and *TNF*α was impaired in *A2B1^−/−^* bone marrow-derived macrophages (BMDMs), isolated from conditional *A2B1^fl/fl^Lyz2-Cre^–/–^* mice (Fig. S2A). To further determine the role of A2B1 in antiviral immunity, secretion levels of IFNβ were detected in THP-1 and MEF cells via enzyme-linked immunosorbent assay (ELISA). Our data showed that the production of IFNβ was decreased remarkably in *A2B1^−/−^* THP-1 and MEF cells throughout the entire course of SFTSV infection (Fig. 2D). In addition, we observed that protein levels of p-TBK1 and p-IRF3 were significantly decreased in A2B1-deficient THP-1 and MEF cells (Fig. 2E and F), and further verified by quantitative analysis (Fig. 2G and H). Besides, TCID_50_ was used to detect the exocellular viral titers. Consistent with the results of A2B1 knockdown, SFTSV titers were decreased in *A2B1^−/−^* THP-1 and MEF cells (Fig. 2I and J). These results illustrate that A2B1 should be involved in the activation of the IFN-relevant antiviral signaling pathway during SFTSV infection.

### SFTSV NP is important for the retention of A2B1 to interact with the STING-TBK1 axis

**Fig 3:**
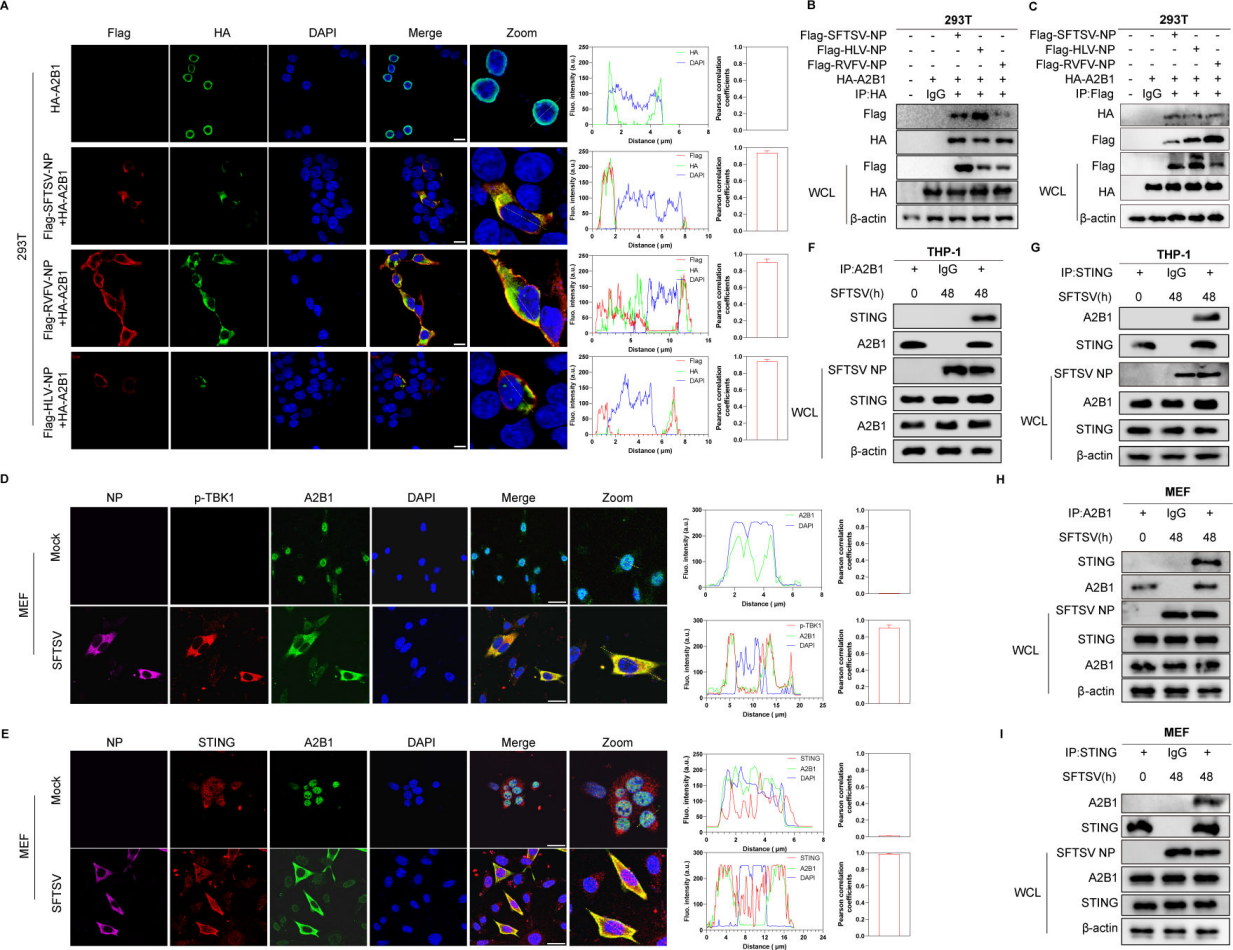
NP is important for the retention of A2B1 to interact with the STING-TBK1 axis. (**A**) HEK293T cells were transfected with Flag-tagged SFTSV NP, Flag-tagged RVFV NP, Flag-tagged HLV NP, and HA-tagged A2B1 for 24 h, and SFTSV NP (red), RVFV NP (red), HLV NP (red), A2B1 (green), and DAPI (blue) were analyzed by confocal microscopy. Fluorescence intensity analysis and the Pearson’s correlation coefficient calculation were performed by ImageJ software. Scale bar: 5 µm. (**B and C**) HEK293T cells were transfected with the indicated plasmids for 24 h, and interaction between HA-tagged A2B1 and Flag-tagged SFTSV NP, Flag-tagged HLV NP, and Flag-tagged RVFV NP was detected by Co-IP. (**D and E**) MEF cells were infected with or without SFTSV (MOI = 10) for 48 h, SFTSV NP (purple), p-TBK1 (red) (**D**), STING (red) (**E**), A2B1 (green), and DAPI (blue) were analyzed by confocal microscopy. Fluorescence intensity analysis and the Pearson’s correlation coefficient calculation were performed by ImageJ software. Scale bar: 10 µm. (**F–I**) THP-1 (**F and G**) and MEF (**H and I**) cells were infected with or without SFTSV (MOI = 10) for 48 h, and interaction between A2B1 and STING in THP-1 and MEF cells was analyzed by Co-IP.

A previous study showed SFTSV NP was important for the translocation of A2B1 (34). In our results, we observed that A2B1 was totally expressed in the nucleus when transfected alone. Nevertheless, A2B1 appeared in the cytoplasm when co-transfected with SFTSV NP (Fig. 3A), suggesting the nucleocytoplasmic translocation of A2B1 was mediated by SFTSV NP. A similar phenomenon was also observed in HEK293T cells overexpressed with Rift Valley fever virus (RVFV) NP or Heartland virus (HLV) NP (Fig. 3A). The NP sequences from these three bunyaviruses are conserved; all of them could participate in the interaction with A2B1. The co-immunoprecipitation (Co-IP) assays further confirmed the interaction between A2B1 and bunyavirus NP from SFTSV, HLV, or RVFV, respectively (Fig. 3B and C). It is known that bunyavirus NP is associated with the retention of hnRNP U. Previous studies showed that STING plays a key role in the induction of the type I IFN signaling pathway during SFTSV infection. In addition, the STING-TBK1 axis is required for mediating the activation of A2B1 via interacting with A2B1 in the cytoplasm directly (20). Hence, to detect whether STING-related signaling could mediate the activation of A2B1 during SFTSV infection, the interaction between the STING-TBK1 axis and A2B1 was detected by confocal microscopy and Co-IP assays during SFTSV infection. Our confocal microscopy results showed that A2B1 was colocalized with p-TBK1 and STING in the cytoplasm after SFTSV infection (Fig. 3D and E). Furthermore, the endogenous A2B1 and STING could be mutually pulled down in THP-1 and MEF cells under SFTSV infection (Fig. 3F through I). Those results demonstrate that A2B1 could be retained by SFTSV NP in the cytoplasm and interact with STING directly and mediate the activation of antiviral innate immune responses during SFTSV infection.

### A2B1 initiates an anti-SFTSV immune response by recognizing mtDNA

**Fig 4:**
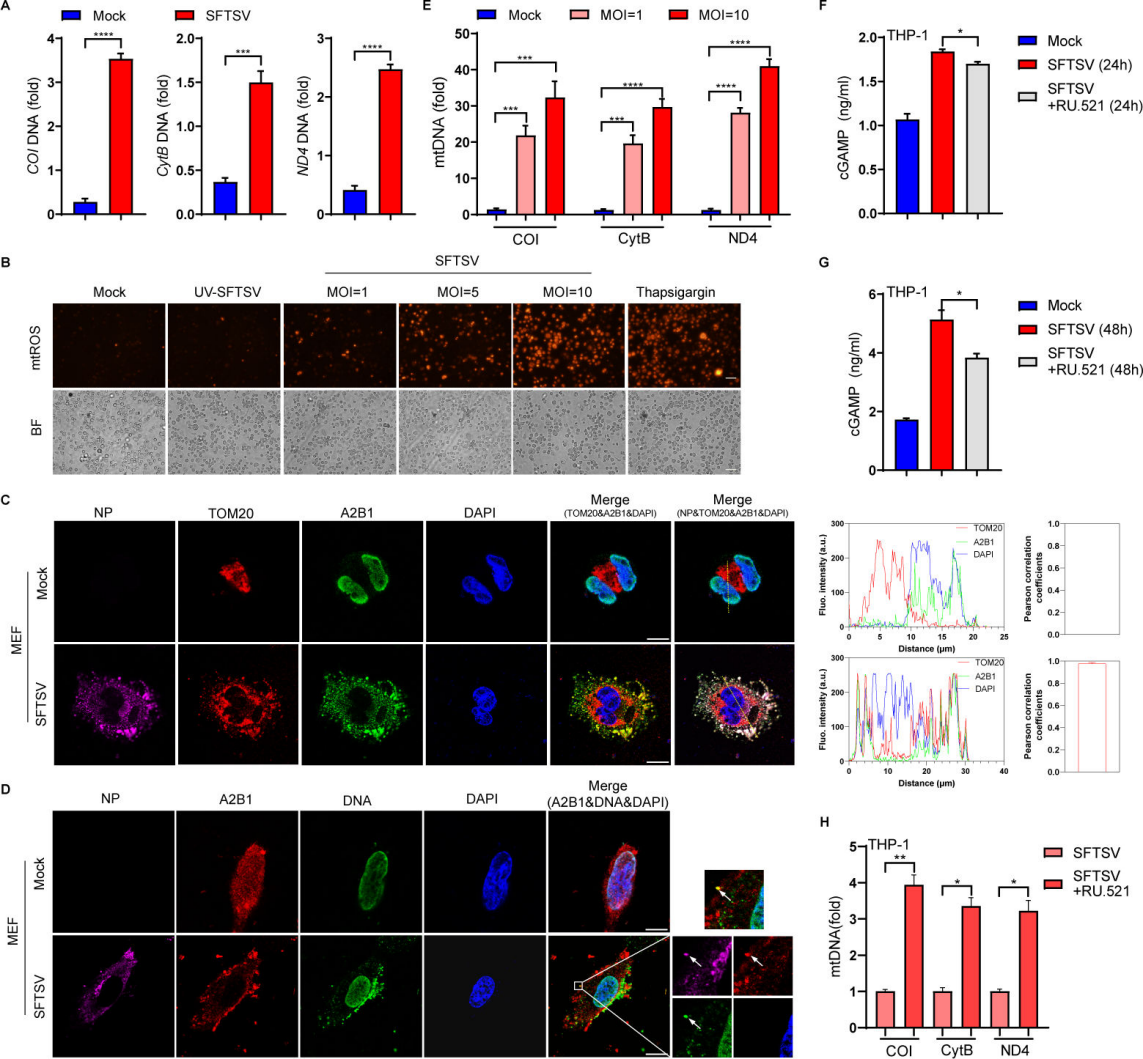
A2B1 is capable of recognizing mitochondrial DNA during SFTSV infection. (**A**) THP-1 cells were infected with or without SFTSV (MOI = 10) for 48 h, total DNA was extracted from the cell lysate, and the released mtDNA (*COI*, *CytB,* and *ND4*) was quantified by qPCR. (**B**) THP-1 cells were infected with UV-inactivated SFTSV or SFTSV (MOI = 1, 5, and 10) for 48 h, or treated with thapsigargin for 6 h. Intracellular mtROS was stained with MitoSOX (red). BF, bright field. Scale bar: 200 µm. (**C**) MEF cells were infected with SFTSV for 48 h, and SFTSV NP (purple), TOM20 (red), A2B1 (green), and DAPI (blue) were analyzed by confocal microscopy. Fluorescence intensity analysis and the Pearson’s correlation coefficient calculation were performed by ImageJ software. Scale bar: 10 µm. (**D**) MEF cells were infected with SFTSV for 48 h, and SFTSV NP (purple), A2B1 (red), DNA (green), and DAPI (blue) were analyzed by confocal microscopy. Images on the right indicated the square zoom in each panel. Arrows mark the colocalization of A2B1 with SFTSV NP and DNA. Scale bar: 10 µm. (**E**) THP-1 cells were infected with or without SFTSV (MOI = 10) for 48 h, and total DNA was immunoprecipitated by A2B1, and mtDNA (*COI*, *CytB,* and *ND4*) levels were quantified by qPCR. (**F and G**) Mock-treated, SFTSV-infected, and SFTSV-infected with RU.521 (20 μM)-treated THP-1 cells at 24 h (**F**) or 48 h (**G**) were processed, and the cytoplasmic cGAMP was measured by ELISA. (**H**) THP-1 cells were infected with SFTSV (MOI = 10) in the absence or presence of RU.521 (20 μM) for 48 h, total DNA was immunoprecipitated by A2B1, mtDNA (*COI*, *CytB,* and *ND4*) levels were quantified by qPCR. Data were obtained from three independent experiments (*n* = 3). *P < 0.05, **P < 0.01, ***P < 0.001, ****P < 0.0001.

Mitochondria are ubiquitous cellular organelles. Importantly, accumulated studies have revealed the important role of mitochondria in mediating antiviral innate immunity (28, 35–37). Under virus infection, mitochondrial stress could promote the release of mitochondrial damage-associated molecular patterns (DAMPs), mitochondrial DNA (mtDNA), which is now accepted as a stimulator of the host antiviral system. Many studies indicate that mislocalized mtDNA is an important DAMP that could activate multiple cytoplasmic DNA sensors to initiate antiviral immunity (28, 38–40). Based on our results above, we hypothesized that cytoplasmic A2B1 could serve as a DNA sensor involved in the recognition of mtDNA during SFTSV infection. Next, we detected the mislocalized mtDNA in the cytoplasm and the production of mtROS that could indicate the dysfunction of mitochondria (41, 42). Interestingly, we found that the released mtDNA was significantly increased in THP-1 cells during SFTSV infection (Fig. 4A). Moreover, our results showed that the live SFTSV, but not the UV-inactivated SFTSV, could promote the production of mtROS in THP-1 cells in a SFTSV dose-dependent manner (Fig. 4B). Thapsigargin was used as a positive control to induce mitochondrial dysfunction (43). To further verify whether the mtDNA is involved in triggering the activation of A2B1 during SFTSV infection, confocal microscopy was used to detect the colocalization between mitochondria, mtDNA, and A2B1. TOM20, the mitochondrial outer membrane marker, was observed to determine the spatial position of A2B1 and mitochondria. Our results showed that A2B1 was translocated to mitochondria, showing colocalization with mitochondria (Fig. 4C) and cytoplasmic DNA (Fig. 4D) in MEF cells during SFTSV infection. To further determine the direct interaction between A2B1 and mtDNA under SFTSV infection, mtDNA was detected from A2B1 pulled-down production by qPCR. Our results showed an average enrichment of at least 20-fold for mtDNA fragments tested in THP-1 cells during SFTSV infection (Fig. 4E). Moreover, to confirm the roles of SFTSV NP and mtDNA in the activation of the STING-TBK1 signaling pathway by A2B1, MEF cells were transfected with SFTSV NP and mtDNA respectively or simultaneously. We found the level of *IFN*β transcription was increased with mtDNA transfection; however, overexpression of SFTSV NP alone barely induced *IFN*β transcription, but SFTSV NP had a synergistic effect on mtDNA in innate immunity (Fig. S3A). Besides, confocal microscopy results showed that A2B1 was colocalized with STING in the cytoplasm when SFTSV NP existed; nevertheless, the nucleoplasmic translocation of A2B1 could not be observed with mtDNA overexpression (Fig. S3B and C). When mtDNA was co-transfected with SFTSV NP, A2B1 could be colocalized both with STING and p-TBK1 in the cytoplasm (Fig. S3B and C). These results further suggested that the combination of SFTSV NP and A2B1 in the cytoplasm created an opportunity for A2B1 to recognize mtDNA, thereby activating the STING-TBK1 signaling pathway.

It has been reported that cytoplasmic mtDNA binds to cGAS and activates the cGAS-STING signaling pathway under SFTSV infection (25). To illustrate the precise role of A2B1 and cGAS in sensing mtDNA for STING-TBK1 activation, we utilized the cGAS inhibitor RU.521 under SFTSV infection and measured the cytoplasmic cGAMP with ELISA. Our data showed that the cytoplasmic cGAMP levels in SFTSV-infected THP-1 cells were 1.7- to 5-fold higher than uninfected cells, and the production of cGAMP was partially inhibited with RU.521 existence (Fig. 4F and G), suggesting that the cGAS-STING signaling pathway can be activated after SFTSV infection. Furthermore, we performed DNA immunoprecipitation combined with qPCR to detect the interaction between A2B1 and mtDNA, with RU.521 existing. We found that more mtDNA (*COI*, *CytB*, and *ND4*) was pulled down by A2B1 when cGAS was blocked (Fig. 4H), suggesting A2B1 may have a competitive relationship with cGAS in binding to mtDNA. Those data indicate that SFTSV infections cause mitochondrial dysfunction and mtDNA release. A2B1 could recognize cytoplasmic mtDNA directly and mediate antiviral immunity.

## DISCUSSION

SFTSV, recently named *Dabie bandavirus*, is a newly identified bunyavirus, posing a serious threat to public health. SFTSV activates antiviral innate immune responses through interactions with PRRs, including RIG-I, SAFA, and cGAS, to induce the production of type I IFNs and proinflammatory cytokines. Although there are no specific therapies for SFTS, and the underlying pathogenic mechanism of SFTSV is poorly understood (44), the roles of RNA and DNA sensors in the recognition and resistance of SFTSV infection have been studied and may provide strategies for the treatment of SFTS.

A2B1 is a member of the hnRNPs family and has been recently identified as a nuclear DNA sensor. In the present study, we found that during RNA virus SFTSV infection, A2B1 was detained by SFTSV NP in the cytoplasm and recognized mtDNA to mediate IFN-dependent signaling activation. A2B1 is an important regulator of antiviral innate immunity. Upon sensing viral DNA in the nucleus, A2B1 is demethylated by JMJD6 and dimerizes, which results in the cytoplasmic translocation. The cytoplasmic A2B1 interacts and activates the STING-TBK1-IRF3 signal transduction cascade, facilitating the transcription of IFN-Is to resist DNA virus HSV-1 invasion (20). Furthermore, A2B1 protein is highly expressed during EV-71 infection and is redistributed from the nucleus to the cytoplasm. After inhibition of A2B1 expression, EV-71 replication in SK-N-SH cells is effectively inhibited (45). Recently, PAC5 has been found to be a small-molecule agonist of A2B1 for the inhibition of HBV and SARS-CoV-2 omicron infection. PAC5 binds to A2B1, promotes formation of the A2B1 dimer and nucleocytoplasmic translocation, resulting in activation of TBK1-IRF3 signaling and promotion of STAT1/2 phosphorylation (46). In addition, many other hnRNPs are shown to be involved in innate immune response (47). hnRNPK can enhance the expression of myeloid differentiation primary response 88 (MyD88), subsequently regulating the phosphorylation of TBK1 and IRF3, activating the IFN signaling pathway to promote antiviral immunity (48). hnRNPE1 acts as a cofactor for the recognition of cytoplasmic DNA by cGAS, promoting the binding of cGAS to DNA. Thus, hnRNPE1 enhances cGAS activation and downstream signaling to insist on HSV-1 invasion (49). The host utilizes mechanisms to regulate immune response to resist viruses' invasion, but viruses can hijack these mechanisms and exploit them for their benefit, resulting in negative regulation of innate immunity (47). It is known that A2B1 contributes to multiple aspects of RNA metabolism (23, 50). Some studies are reporting the roles of A2B1 in virus replication and viral RNA synthesis. The A2B1 has been shown that during SARS-CoV-2 infection, A2B1 can be bound by viral NSP1 protein directly in the cytoplasm, leading to restrained immune response and enhanced infection by SARS-CoV-2 and other β-coronaviruses (51). Besides, A2B1 can interact with the 5′ UTR of SFTSV RNA and upregulate the replication of RNA viruses including JEV, SFTSV, VSV-GFP, SeV, EV71, and ZIKV (34, 52).

A2B1 seems to play dual roles. On the one hand, A2B1 acts as a PRR to recognize pathogenetic DNA for the initiation of antiviral immune response. On the other hand, A2B1 can be hijacked in its cellular localization by viral proteins or utilized by the virus for replication to limit antiviral innate immunity. The virus–host relationship is a dynamic arms race in which each attempts to suppress the other (47). Although the RNA virus SFTSV seizes A2B1 for its own RNA synthesis, A2B1 also contributes to the host antiviral immune response as a PRR, undeniably. Here, we observed that SFTSV NP bound A2B1, promoting A2B1 nucleocytoplasmic translocation. Similar results were also observed with RVFV NP and HLV NP. The interaction between A2B1 and STING/p-TBK1 in the cytoplasm demonstrated the role of A2B1 in mediating the activation of the STING-TBK1 signaling pathway to promote IFNβ secretion. We found that the transcription and protein secretion levels of IFNβ were reduced remarkably when *A2B1* was silenced, similar to the expression levels of p-TBK1 and p-IRF3. Although after knockdown or knockout of A2B1, SFTSV titers were decreased, the powerful combination between A2B1 and NP/STING/p-TBK1/p-IRF3/mtDNA confirmed the activation of DNA sensor A2B1 in innate immune response. Given the previous report, it has been shown that A2B1 can promote the replication of SFTSV. Our data illustrate that A2B1 is capable of performing the function of DNA sensor during SFTSV infection, suggesting A2B1 may play a promoting role in the net impact of SFTSV infection. However, we cannot deny the antiviral function exerted by A2B1.

Mitochondria are ubiquitous cellular organelles, and their homeostasis is typically associated with the accumulated ROS and mislocated mtDNA in the cytoplasm (53). Of note, mislocalized mtDNA could be recognized by different PRRs such as cytoplasmic cGAS, NLRP3, and endosomal TLR9 to initiate antiviral innate immunity (27, 28). In our study, we found that SFTSV infection could induce mitochondrial stress and promote the release of mtDNA in the cytoplasm. Importantly, apart from recognizing pathogenic DNA, A2B1 was confirmed to recognize and bind mislocalized mtDNA during SFTSV infection, leading to interaction with the STING-TBK1 axis. SFTSV NP is the key for A2B1 nucleoplasmic translocation, creating an opportunity for A2B1 to recognize mtDNA. The combination of A2B1 and mtDNA activates the novel function of A2B1 in antiviral immunity. Previous studies verified that the mechanism of cGAS recognizing SFTSV was by cGAS interacting with mislocated mtDNA in the cytoplasm (25). The mechanism by which the novel DNA sensor A2B1 surveilled the invasion of RNA virus SFTSV was also through the recognition of mtDNA. Whether cGAS and A2B1 have a cooperative and competitive relationship in the recognition and binding of mtDNA needs further investigation. Our results extended the way A2B1 can be activated. Not only DNA viruses, but also RNA viruses can be sensed by A2B1. The change in subcellular localization of A2B1 caused by SFTSV infection is necessary for the recognition of mtDNA.

**Fig 5:**
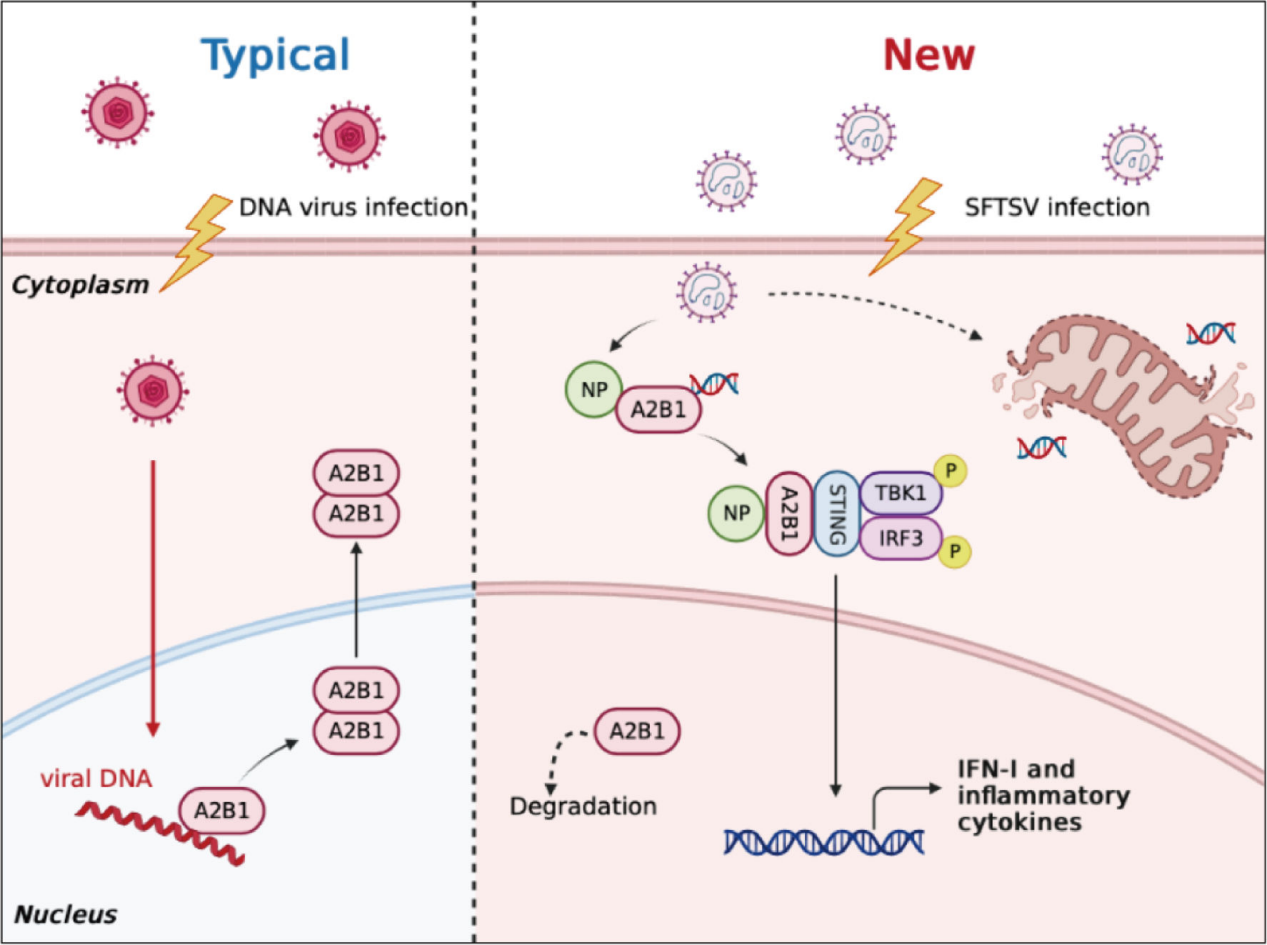
Schematic model illustrating the role of A2B1 in sensing RNA virus SFTSV infection via mtDNA. In the canonical pathway, A2B1 recognizes the viral DNA in the nucleus, leading to nucleocytoplasmic translocation. In our study, we found that after RNA virus SFTSV infection, the newly synthesized A2B1 in the cytoplasm was captured by SFTSV NP and remained in the cytoplasm, recognizing mtDNA released by the damaged mitochondria, thus activating the STING-TBK1 signaling pathway and promoting the secretion of type I interferon.

In conclusion, our study has investigated the antiviral mechanism of nuclear DNA sensor A2B1 in response to the RNA virus SFTSV infection. Our results expand new knowledge on the role of A2B1. Upon SFTSV infection, A2B1 was hijacked by SFTSV NP in the cytoplasm. After sensing and binding mislocated mtDNA, which was from dysfunctional mitochondria, A2B1 was activated to promote IFNβ-dependent antiviral immunity (Fig. 5). Thus, A2B1 is the novel DNA sensor that can recognize DNA virus directly in the nucleus, either sense RNA virus indirectly in the cytoplasm through mtDNA. Our findings open up a new way for studying host defense against RNA viruses. More intensive future efforts are warranted to fully investigate the underlying roles of A2B1 during virus infection.

## MATERIALS AND METHODS

### Cells, mice, viruses, and plasmids

Vero, MEF, and HEK293T cells were cultured in DMEM medium (Gibco, Beijing, China) containing 10% fetal bovine serum (FBS; Gibco, Auckland, New Zealand) and 1% streptomycin-penicillin (p/s) at 37°C with 5% CO_2_. THP-1 cells were maintained in RPMI 1640 medium (Gibco, Beijing, China) supplemented with 10% FBS (Gibco, Auckland, New Zealand), and induced with phorbol 12-myristate 13-acetate (PMA; 100 ng/mL) for 48 h to promote cell differentiation. After treatment for 24 h, THP-1 cells were replaced with new medium without PMA.

The *A2B1*^fl/fl^ and conditional *A2B1*^fl/fl^*Lyz2-Cre^–/–^* mice on a C57BL/6J background were kindly provided by Professor Xue-tao Cao from Nankai University in Tianjin, China. The *A2B1* gene was identified with PCR. Mice were bred in pathogen-free conditions. Mouse bone marrow-derived macrophages (BMDMs) from *A2B1*^fl/fl^*Lyz2-Cre^–/–^* mice were isolated from mouse (6–10 weeks of age) hind legs, using RPMI 1640 medium to flush marrow from femurs into a 10 cm dish. Then add 10 ng/mL M-CSF (macrophage colony stimulating factor), which is an essential regulator of monocyte/macrophage proliferation to differentiation and survival.

SFTSV (strain JS2011-013-1) was cultivated in Vero cells at an MOI of 0.01. The culture medium was changed to DMEM containing 2% FBS after infection for 2 h, and the collected cellular supernatant 6–7 days post-infection, freezing and thawing twice for cell disruption, was stored at −80°C.

pCDNA3.1(+) plasmids encoding Flag-SFTSV-NP, Flag-HLV-NP, Flag-RVFV-NP, and HA-A2B1 were constructed and synthesized by Sangon Biotech (Shanghai, China).

### RNA interference and the CRISPR/Cas 9 system

The siRNA duplexes were transfected into THP-1 and MEF cells using Lipofectamine RNAiMAX (Invitrogen, Carlsbad, CA) to knock down A2B1. The target sequences used for transient silence were as follows: 5′-CTTTGGTGGTAGCAGGAAC-3′ for human A2B1, 5′-GAGGAAATTATGGAAGTGG-3′ for mouse A2B1, and 5′-TTCTCCGAACGTGTCACGT-3′ for negative control.

*A2B1*^−/−^ THP-1 and MEF cell lines were generated using the CRISPR/Cas9 system, and the gene-specific single-guide RNA (sgRNA) sequence was designed by the online CRISPR Design Tools. The sgRNA sequences were as follows: 5′-CACCGGTTCCTCAAACTTTCTTCTG-3′ for human A2B1, and 5′-CACCGGGAATGGGGCCTTGCAGCCA-3′ for mouse A2B1. In brief, after LentiCRISPRv2-A2B1, pMD2.G and psPAX2 were packaged together with polyetherimide (PEI) and co-transfected into HEK293T for 48–60 h. The supernatant was collected for precipitation with PEG8000, and the resuspended lysate was mixed with THP-1 cells previously containing 5 g/mL polybrene. The selected clonal cells were identified by gene sequencing and Western blot.

### Antibodies

Primary antibodies specific for hnRNPA2B1 (31607) and STING (19851-1-AP) were obtained from Proteintech (Wuhan, China); primary antibodies specific for β-actin (GB15001-100), HA-tag (GB12939-100), and Flag-tag (GB15939-100) were obtained from Servicebio (Wuhan, China); primary antibodies specific for TBK1 (38066), p-TBK1 (Ser172) (5483), and TOM20 (42406) was obtained from Cell Signaling Technology (Beverly, MA); primary antibodies specific for IgG (550017), IRF3 (R26922), p-IRF3 (Ser386) (R381561), and HA-tag (250113) were obtained from Zenbio (Chengdu, China); primary antibodies specific for Flag-tag (ABT2010) were obtained from Abbkine (Wuhan, China); anti-DNA (ABE2601) antibody was obtained from Merck Millipore (Darmstadt, Germany); and the antibody specific to SFTSV NP was maintained in our laboratory.

### Cell proliferation assay

Cell viability was assessed using the Cell Counting Kit-8 (CCK-8; Servicebio, Wuhan, China) following the manufacturer’s instructions.

### Western blot analysis

The cell lysate was diluted with RIPA lysis buffer (Beyotime, China), briefly ultrasonicated, and stored at −80°C. The protein samples were heated at 95°C for 10 min and separated by 12% SDS-PAGE, transferred to polyvinylidene difluoride (PVDF) membranes (Millipore, USA), blocked with 5% non-fat milk Tris-buffered saline and Tween 20 (TBST) for 1 h, and incubated with corresponding primary antibodies overnight at 4°C, followed by incubation with secondary antibodies for 1 h and washing with PBST. The protein bands were detected using the ChemiDoc touch imaging system (Bio-Rad) and processed using ImageLab software.

### RNA extraction and qPCR

RNA was isolated using RNA extraction solution (Servicebio, Wuhan, China). The 2 × 10^6^ cells were lysed in 0.5 mL RNA extraction solution. The lysate was added with 0.5 mL of chloroform, shaken thoroughly, incubated for 3 min, centrifuged for 10 min at 12,000 × *g* at 4°C; the supernatant was added with 0.5 mL of isopropanol and centrifuged under the same conditions after incubating for 10 min. The RNA was washed with 75% ethanol, and the total RNA was dried in air.

The cDNA was synthesized using the High-Capacity cDNA Reverse Transcription Kit (Invitrogen, Carlsbad, CA). The qPCR was performed with specific primers listed in Table S1. Relative mRNA concentrations were calculated by the 2^−ΔΔCt^ method, normalizing with β-actin.

### Confocal microscopy

MEF cells were infected with SFTSV (MOI = 10) for 48 h. MEF or HEK293T cells were transfected with indicated plasmids or mtDNA for 24 h. The cell samples were fixed in 4% (wt/vol) paraformaldehyde for 20 min, permeabilized with 0.2% Triton X-100, blocked in 5% bovine serum albumin (BSA) for 30 min, incubated with corresponding primary antibodies overnight at 4°C, and labeled with fluorescently secondary antibodies for 1 h. Nuclei were stained with 4′,6-diamidino-2-phenylindole (DAPI; Beyotime, Shanghai, China) for 15 min. The cell samples were observed using an Olympus FV3000RS confocal laser microscope with either a 60× or 100× objective lens. Two co-localized fluorescence signals were analyzed using ImageJ 1.0 software.

### Co-immunoprecipitation

To confirm the interaction of indicated proteins, cells were infected with SFTSV for 48 h or transfected with appropriate plasmids for 24 h for co-immunoprecipitation (Co-IP). The cells were lysed with IP cell lysis buffer (Beyotime, China), incubated with specific antibody or IgG as negative control overnight at 4°C. Protein A+G agarose (Beyotime, China) was added and gently rotated at 4°C for 3 h. The mixture was then centrifuged and washed five times with PBS. The beads were collected and resuspended with SDS-PAGE loading buffer and analyzed by Western blot.

### Detection and quantification of mtDNA

THP-1 cells were infected with or without SFTSV (MOI = 10) for 48 h, and total DNA was extracted from cell lysate using QIAamp DNA Mini Kit (Qiagen, Hilden, Germany). The released mtDNA was detected from total DNA by qPCR.

For quantification of mtDNA pulled down by A2B1, THP-1 cells were infected with or without SFTSV (MOI = 10) for 48 h, and the cell lysate was diluted by NP-40 (Cell Signaling Technology, Beverly, MA), incubated on ice for 15 min, centrifuged for 10 min at 16,000 × *g* at 4°C. The cell supernatant was added with antibody of A2B1 or IgG as control, gently rotated overnight at 4°C. Protein A+G Agarose (Fast Flow, for IP) (Beyotime, China) was mixed and incubated at 4°C for 3 h, centrifuged for 5 min at 2,500 × *g*, and washed with PBS for five times, and DNA was from supernatant by QIAamp DNA Mini Kit according to the manufacturer’s instructions. The qPCR was performed to measure the human mtDNA cytochrome c oxidase subunit I (*COI*), cytochrome B (*CytB*), and dehydrogenase subunit 4 (*ND4*) of the DNA binding to A2B1 in the cell extract samples. The expression values of each replicate were normalized against 18S cDNA using the 2^−ΔΔCt^ method.

### Enzyme-linked immunosorbent assay

Quantification of IFNβ and cGAMP was analyzed using IFN-beta Quantikine ELISA Kit (R&D Systems, USA) and 2′3′-cGAMP ELISA Kit (Cayman, USA), respectively.

### Mitochondria isolation

Mitochondria from MEF cells were isolated using the Mitochondria Isolation Kit (Thermo Scientific, USA). Then mtDNA was extracted from isolated mitochondria using QIAamp DNA Mini Kit (Qiagen, Hilden, Germany).

### Statistical analysis

The mRNA and mtDNA levels were analyzed by Student’s *t* test or one-way analysis ANOVA with GraphPad Prism Software, as well as CCK-8 proliferation assays and Western blot quantitative analysis. All the data were based on a minimum of three independent experiments, and the statistical significance was considered with *P* < 0.05

## SUPPLEMENTAL MATERIAL

Fig. S1 mbio.01668-25-s0001.tif The RNAiMAX reagent Lipofectamine and SFTSV infection have no effect on MEF cells viability.

Fig. S2 mbio.01668-25-s0002.tif The mRNA transcription of inflammatory cytokines are reduced in A2B1 deficiency BMDM cells with SFTSV infection.

Fig. S3 mbio.01668-25-s0003.tif The combination of SFTSV NP and A2B1 promotes mtDNA recognition by A2B1 for activation of the STING-TBK1 axis.

Tables S1 mbio.01668-25-s0004.xlsx The primer sequences used for qPCR.

ASM does not own the copyrights to Supplemental Material that may be linked to, or accessed through, an article. The authors have granted ASM a non-exclusive, world-wide license to publish the Supplemental Material files. Please contact the corresponding author directly for reuse.
