# Supplementary figures and images for: hnRNPA2B1 recognizes RNA virus SFTSV infection through mitochondrial DNA
Source: mBio. 2025 Jul 21;16(8):e01668-25. doi: 10.1128/mbio.01668-25 (PMC12345236; doi:10.1128/mbio.01668-25)

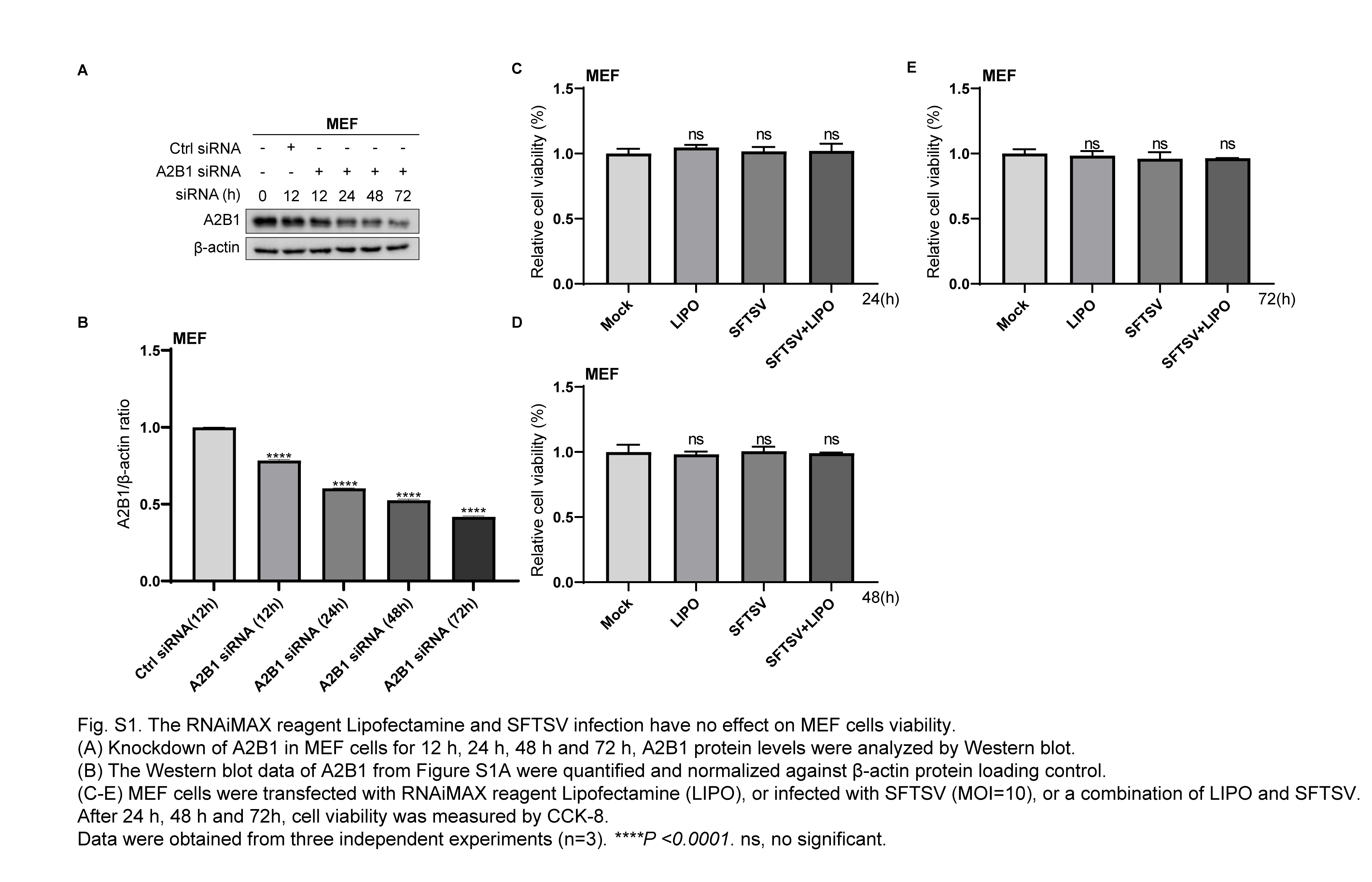

Supplement: Fig. S1 — The RNAiMAX reagent Lipofectamine and SFTSV infection have no effect on MEF cells viability. [file mbio.01668-25-s0001.tif]

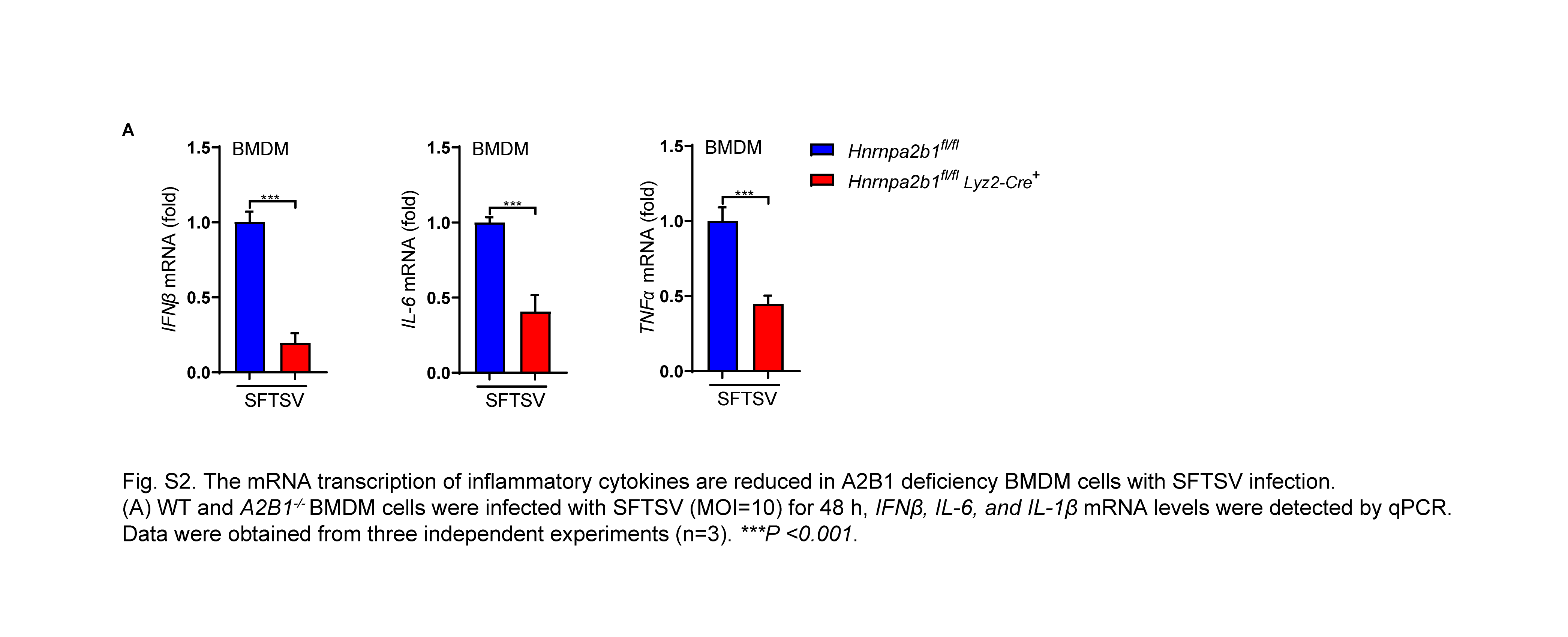

Supplement: Fig. S2 — The mRNA transcription of inflammatory cytokines are reduced in A2B1 deficiency BMDM cells with SFTSV infection. [file mbio.01668-25-s0002.tif]
